# AI-driven biomaterial design: an intelligent closed loop from reverse design to biological response

**DOI:** 10.3389/fcell.2025.1755565

**Published:** 2026-01-05

**Authors:** Minglei Liu, Yichuan Zhou, Xiaohan Mei, Zehao Yu, Boyun Guan, Yi Xiao, Shixian Liu, Hao Wang, Yanguo Qin

**Affiliations:** 1 Department of Orthopedics, The Second Hospital of Jilin University, Changchun, China; 2 Key Laboratory of High Performance Plastics, Ministry of Education, College of Chemistry, Jilin University, Changchun, China; 3 Joint International Research Laboratory of Ageing Active Strategy and Bionic Health in Northeast Asia of Ministry of Education, Jilin University, Changchun, China

**Keywords:** artificial intelligence, biomaterial design, deep learning, machine learning, performance prediction

## Abstract

Traditional approaches to biomaterial design face numerous challenges, including high trial-and-error costs, long development cycle, and the difficulty in deciphering the complex relationship between material properties and biological responses. With the rise of artificial intelligence (AI) technology, its capabilities in processing high-dimensional data and constructing complex mapping relationships have brought revolutionary changes to biomaterial design. This article reviews the four core applications of AI in the design of biomaterials. Firstly, based on the therapeutic needs of diseases, the functions of materials are clarified and formulations are generated. Secondly, high-throughput prediction and virtual screening of material properties using AI models significantly reduce development costs. Furthermore, the performance of materials and production efficiency can be enhanced by optimizing material formulas and processing techniques through AI. Finally, AI is used to predict the interaction between materials and cells or tissues, and to assess the safety and efficacy of the materials. This paper systematically explores how AI empowers biomaterial design, driving its advancement toward precision and intelligence, thereby providing robust support for the realization of personalized and precision medicine.

## 1 Introduction

Biomaterials are special functional materials, which can be natural or man-made. They interact with living systems and include metallic, inorganic, and organic types (Zhang et al., 2025b). In modern medicine, biomaterials are key for medical devices, tissue engineering scaffolds, and drug delivery systems (Liu et al., 2023; Zhou et al., 2024). Clinically, they are used in biodegradable polymer catheters, bone implants, skin grafts and so on (Song et al., 2024; Wang et al., 2024b; Wang et al., 2025c). These advanced biomaterials, with stable physical and chemical properties and controlled drug release, have advanced regenerative and precision medicine.

However, despite high demand and wide use, the traditional way of developing biomaterials is facing bigger problems. Creating biomaterials has usually relied on researchers’ skills, trial-and-error methods, or accidental discoveries during experiments. This process is slow and costly, greatly limiting the improvement of existing biomaterials and the creation of new ones (Yang et al., 2021). These challenges affect all stages of biomaterial development, including designing materials that meet therapeutic needs, predicting their performance, optimizing production processes, and predicting and evaluating the interaction between materials and organisms.

First of all, at the stage of biomaterial design that is consistent with the treatment goal, researchers will customize materials to meet certain clinical needs, such as promoting angiogenesis or inhibiting the growth of specific bacteria. However, traditional methods often lack systematic design principles. For example, when developing soluble microneedles for the treatment of androgenic hair loss, researchers have to rely entirely on empirical knowledge and rigorous experiments to evaluate the combination of polymers, crosslinking agents and drug vectors to meet the requirements of high hardness and rapid release at the same time (Sun et al., 2024; Wang et al., 2022).

Secondly, in the stage of predicting material properties, the physical, chemical and mechanical properties of biological materials, such as degradation rate, conductivity and viscosity, are the key to their successful application. In the past, these properties had to be obtained through tedious synthesis and characterization processes. To predict the diameter and strength of electrospun fibers, researchers repeatedly adjust parameters such as polymer concentration and voltage, followed by electron microscopy observation and mechanical testing of each sample. This process yields only a vague correlation between process conditions and properties, making precise prediction prior to fabrication impossible (Cho et al., 2025; Wang et al., 2024a).

Furthermore, during the stage of optimizing material preparation processes, especially in advanced manufacturing technologies such as 3D printing and microfluidic technology, even minor changes in process parameters such as printing speed and temperature significantly affect the quality of the final product (Zheng et al., 2024). Traditional optimization methods, such as single-factor experimental design, struggle to handle the complex interactions among multiple parameters. To get a methyl acrylate-modified gelatin (GelMA) hydrogel with the desired stiffness, researchers often need to conduct extensive experiments, exploring different parameter combinations like photoinitiator concentration and exposuwre time (Zhu et al., 2024). This procedure is inefficient and it is difficult to produce the desired biomaterials.

Finally, in the phase of forecasting biomaterial-biological interactions, researchers assess the material’s biocompatibility, immunogenicity, and biological functions. This phase is mainly dependent on many costly *in vitro* cell experiments and *in vivo* animal studies. For example, to evaluate how a nanostructure influences macrophage polarization, conventional methods can only demonstrate the functionality of a specific biomaterial within particular cell or animal models. They fail to predict interactions with complex biological systems during the initial stages of material design, becoming the most significant block in clinical translation (Jia et al., 2025; Wang et al., 2025b).

In this situation, artificial intelligence (AI), particularly machine learning (ML) and deep learning (DL), offers groundbreaking ways to solve the problems. AI refers to technology that mimics human thinking through computer algorithms. AI performs tasks that usually require humans to complete, such as learning, reasoning, problem-solving, visual recognition, etc. (Lehman, 2025). AI uses insights from large amounts of experimental data and existing research to uncover complex relationships between a biomaterial’s structure and its activity. In stark contrast to traditional trial-and-error approaches, AI offers core advantages including the ability to process high-dimensional data, uncover intricate structure-property relationships, and drastically reduce development time and cost through virtual screening and optimization. This shifts biomaterial creation from a trial-and-error approach to a data-based, rational design method. AI can find new material formulas that meet specific performance goals, quickly assess thousands of potential materials in a virtual setting, independently find the best ways to make and process them, and even predict long-term biological effects by looking at early cell shapes (Hang et al., 2025). Within the AI toolkit, different technologies offer complementary strengths: ML excels at extracting design rules from existing datasets, DL unlocks the prediction and generation of complex structures from images or sequences, and generative AI enables the *de novo* creation of materials tailored to multifunctional clinical needs.

**SCHEME 1:**
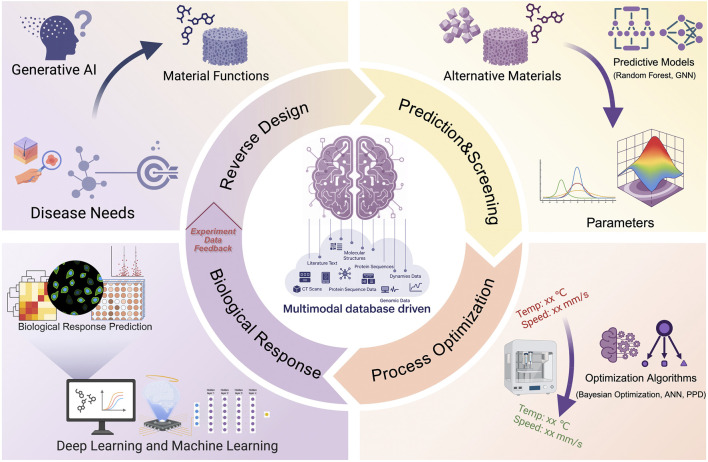
A scheme demonstrating the comprehensive integration of AI technology into the biomaterial design paradigm, including reverse design, prediction and screening, process optimization and biological response.

This review provides a unique perspective by synthesizing these disparate AI methodologies into a coherent, intelligent closed-loop framework—spanning from reverse design and performance prediction to process optimization and biological response evaluation—thereby charting a systematic roadmap for the next-generation of biomaterials (Scheme 1). We will summarize the features of various AI models and their application scenarios in the design of biomaterials (Table 1). Finally, we will look at the current challenges related to data quality and model interpretability, while also looking ahead to future paths that could offer new ideas and concepts for pushing forward the clinical use of smart biomaterials.

**TABLE 1:** The main characteristics and specific applications of different AI methods in the design of biomaterials.

Core technologies	Typical models	Advantages	Disadvantages	Applicable scenarios	Ref.
ML	Ensemble learning	Establishing a quantitative relationship from components or processes to performance	Relatively high computing resources and time costs	Prediction of the mechanical properties of macroscopic materials such as hydrogels and bone scaffolds by the XGBoost model	Ege and Boccaccini (2024), Xu et al. (2025)
				Evaluating the printability of 3D bioprinting ink by RF model	Chen et al. (2023)
				Prediction of the kinetics of drug release in porous polymer carriers by the gradient boosting regression model	Alshahrani et al. (2024)
	Gaussian process regression	Uncertainty quantification and few-shot learning	High computational complexity and difficulty in handling high-dimensional data	Prediction of the drug release curve of acetylated glucan nanofibers by the Gaussian process regression model	Woodring et al. (2025)
				Rapidly identifying topological structures that induced macrophage polarization by Gaussian process regression ML model	Hou et al. (2024)
	Support vector machine	Ability of handling high-dimensional data and strong generalization ability	Low efficiency in training with large-scale samples, and high sensitivity to noise	Prediction of the Young’s modulus and ultimate tensile strength of PVA electrospun scaffolds by support vector machine model	Roldán et al. (2024)
				Real-time assessment of cartilage regeneration status by support vector machine model combined with near-infrared spectrum	Sadeesh et al. (2025)
	Conventional neural network	Ability of handling nonlinear problems and automatic feature engineering	“black box” problem and overfitting risk	Optimizing stiffness of the hydrogel by ANN model	Karaoglu et al. (2023)
				Prediction of the cytotoxicity threshold of zinc-based materials by MLP model	Wang et al. (2025a)
DL	DNN	Constructing complex nonlinear mappings, especially for sequences and high-dimensional data	Vulnerability to adversarial samples and catastrophic forgetting	Prediction of the structure and function of proteins or nucleic acid sequences by AptaTrans	Shin et al. (2023)
				Prediction of the ultimate tensile strength of silk fibers from the amino acid sequences by a DNN classification model	Shin et al. (2024)
	CNN	Processing images and spatial data with strong feature extraction capability	Difficulty in understanding the semantic relationships between regions that are far apart in an image	Creating skeletal structures with specific directional elasticity by CNN model	Wang et al. (2023)
				Automatically recognizing and quantifying nanoscale D-banding collagen fiber patterns in atomic force microscopy images	Huang et al. (2025a)
	PINN	Enhancing the generalization and scientific nature of the model to alleviate the “black box” problem	Difficulty in training and high computational cost	Simultaneously calculating the spatial distribution of an organization’s elastic modulus and Poisson’s ratio by PINN model	Kamali et al. (2023)
				Accurately simulating the macroscopic mechanical behavior of 3D printed biomaterials by PINN model	Meynen et al. (2025)
	Diffusion model	Creating brand-new molecules or structures that conform to constraints	Slow generation speed	Generating novel polypeptide sequences by embedding the attention mechanism in the conditional diffusion framework	Liao et al. (2025)
LLM	GPT	Understanding natural language and achieve creative design based on goals	Limitations of reasoning ability	Constructing a database of nanoparticle materials from a vast amount of literature by NanoSafari	Wang et al. (2025f)
				Generating new molecules and formulations for complex clinical needs, such as multifunctional antimicrobial peptide hydrogels by LLM model	Jiang et al. (2025)
Optimization algorithm	Bayesian optimization	Finding the optimal solution with the fewest attempts	Sharply increasing computational cost as the number of iterations grows	Predicting the viscosity of bio-ink precursors under constraints using the Bayesian optimization framework	Xu et al. (2024)
	genetic algorithm	Solving complex optimization problems involving multiple parameters and multiple objectives	Needing a large number of objective function evaluations to converge to a satisfactory solution	Optimizing the performance of palm fruit by genetic algorithm	Samuel et al. (2024)
	Reinforcement learning	Adaptive ability and potential for continuous learning	Unstable training process and unrepeatable results	Optimizing the generated antimicrobial peptide sequence as part of generative AI	Jiang et al. (2025)

Abbreviations: ANN, artificial neural network; DL, deep learning; DNN, deep neural network; GPT, Generative pre-trained transformer; LLM, large language model; ML, machine learning; MLP, multilayer sensor; PINN, physical information artificial neural network; RF, random forest.

## 2 Reversely designing biomaterials for specific clinical applications

The conventional way of developing biomaterials usually means finding uses for materials that already exist. This method is often inefficient and somewhat random. On the other hand, AI-powered reverse engineering starts with specific clinical problems, defines the functional needs biomaterials must meet in the body, and then uses AI to find the best material formulas and structures to meet those needs. In this big change in biomaterial design, ML and DL have played key roles.

### 2.1 ML-based reverse design of biomaterials

The strength of ML resides in its capacity to analyze extensive high-dimensional datasets and uncover concealed patterns, hence facilitating the reverse extraction of biomaterial design principles from a knowledge repository grounded in current literature. A representation of this process is the utilization of generative AI technologies in data extraction.

For example, NanoSafari, an AI assistant, employs its grouped iterative verification information extraction method to automatically analyze nanoparticle synthesis parameters and characteristic information from over 20,000 published biomedical nanoscience literature. It has constructed a database integrated into a generative large language model (LLM) (Wang et al., 2025f). This completes the initial step: building a searchable, organized database using existing knowledge. The database helps researchers quickly extract the essence of a large number of scientific literature and obtain reliable design parameters for nanomaterials.

Based on a structured database, ML models establish accurate mapping relationships between component and performance, achieving more precise reverse screening and design. For example, a user-friendly ML prediction model was developed to design extracellular matrix-mimicking hydrogels with specific rheological properties for 3D bioprinting. This model utilizes click chemical crosslinking and, based solely on limited experimental data, accurately predict the ratio of gelatin to hyaluronic acid in hydrogels that exhibit specific mechanical behaviors, significantly reducing the financial and time costs of trial and error (Cadamuro et al., 2025). Sirtmilarly, natural language processing techniques are also employed to explore broader material field. Transformer is a neural network architecture based on the self-attention mechanism. It processes the entire sequence data in parallel, significantly enhancing the ability of long-range dependency modeling and training efficiency. Generative pre-trained transformer-4 (GPT-4), a LLM combined with representational clustering technology, was used to mine metal-organic frameworks with specific electrical conductance, demonstrating immense potential of LLM (Zhang et al., 2025a).

**FIGURE 1:**
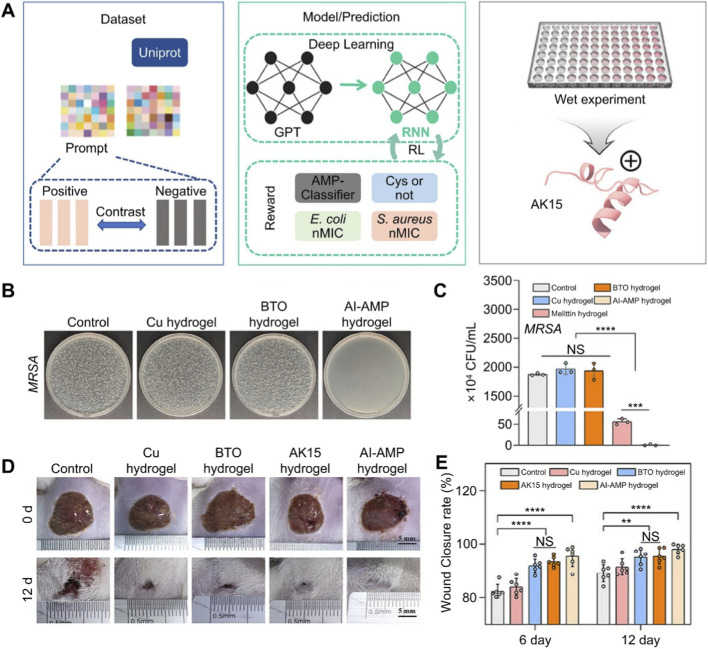
AI-driven automated antimicrobial peptide hydrogel design platform. **(A)** Dataset, prediction model and experimental screening of the platform. **(B)** Representative colony images of MRSA treated hydrogel designed by the platform. **(C)** Colony forming unit of MRSA. **(D)** Wound healing effect of the rats treated by different hydrogels. **(E)** Wound closure rate. Copyright 2025, John Wiley & Sons. Abbreviations: AI-AMP, AI-guided antimicrobial peptide; BTO, Cu-modified barium titanate nanoparticle; CFU, Colony forming unit; GPT, Generative Pre-trained Transformer; MRSA, methicillin-resistant *staphylococcus aureus*; RL, reinforcement learning. All statistical data are represented as mean ± SD (NS: no significance, **P < 0.01, ***P < 0.001, ****P < 0.0001).

When clinical demands become complex and stringent, such as requiring simultaneous fulfillment of multiple functions including antibacterial, good biocompatibility and self-healing property, simple predictive models prove inadequate. To address this issue, Zhang et al. developed an AI-driven automated antimicrobial peptide hydrogel design platform by integrating generative design with multi-objective optimization (Jiang et al., 2025). This platform used cutting-edge ML and AI technologies, such as GPT, prompt-tuning, and reinforcement learning, to generate a novel mercaptan-containing antimicrobial peptide. The AI-guided antimicrobial peptide (AI-AMP) was then functionally coupled with hydrogels to form complex network structures (Figure 1A). AI-AMP achieved a bactericidal efficiency of over 99.99% against methicillin-resistant *staphylococcus aureus* (MRSA) (Figures 1B,C). In a rat model with full-thickness back wound infected with MRSA, AI-AMP achieved a wound healing rate of 99.5% at the end of treatment (Figures 1D,E). The success of AI-AMP marked that AI-based reverse design platforms created unknown biomaterials to meet complex clinical needs.

The advancement of biomaterial reverse design has spurred the development of more sophisticated multi-agent fusion systems. AtomAgents, a generative AI platform synergizes LLM with multiple AI agents specializing in knowledge retrieval, multimodal data integration, and physical simulation. This enables autonomous collaboration of AI agents to tackle difficult material design tasks, like designing metal alloys with superior performance to pure metals (Ghafarollahi and Buehler, 2025).

On the other hand, combining high-throughput experiments with statistical learning provides another powerful path for reverse material design. By combining regression-based statistical learning with high-throughput data acquisition related to gradient surface generation, the optimal conditions beyond the experimental test range could be inferred from the model, and an unprecedented ternary functionalized surface with the best osteogenic, angiogenic and neurogenic activities had been successfully reverse-engineered (Fang et al., 2023). ML also greatly accelerate the reverse exploration process by optimizing the experimental design itself. In the development of microneedles for treatment of androgenetic alopecia, researchers proposed a ML-driven strategy (Yan et al., 2025). By conducting 18 experiments based solely on orthogonal experimental designs, this strategy was able to precisely locate the optimal material composition that simultaneously achieves high hardness and rapid dissolution. This approach combines AI’s predictive power with efficient experimental design, providing a repeatable framework to speed up independent biomaterial research and development.

### 2.2 DL-based reverse design of biomaterials

Unlike traditional ML, DL has unique advantages in processing images, sequences and generating complex unstructured data, thus contributing to the reverse design of biological microstructures.

In the past, the design of porous biological materials used to construct complex structures was limited to regular shapes, such as rod grids. Researchers have introduced the convolutional neural network (CNN) method to explore various random patterns that support biological movement (Wang et al., 2023). CNN uses learnable convolutional kernels to perform local scanning and feature extraction on the input data, thereby efficiently capturing hierarchical patterns in spatial or topological structures. Therefore, CNN model helped create skeletal structures with specific directional elasticity and directly produce large orthopedic implants with the required variable porosity. This method helps to develop new biological materials with unique microstructures.

In short, AI-driven reverse design is changing the field of biomaterial design from an experience-based approach to a rational design method. ML is good at identifying design rules from large data sets and expert knowledge, and can effectively evaluate material combinations and production settings. With its powerful representation and generation capabilities, DL can directly design complex materials with specific structures. The combination of ML and DL provides strong support for the reverse design of biological materials to meet the needs of treatment.

## 3 Predicting the characteristics of biomaterials

Traditional biomaterial characterization relies heavily on advanced technology and repetitive testing, which consumes a lot of manpower, material and financial resources. We use AI to develop algorithm models. These models can predict the final performance based on material composition, structural characteristics or process factors, so as to achieve fast and accurate prediction of biological material properties and promote virtual screening. This section will systematically discuss how to apply AI to predict the structure and physicochemical properties of biological materials from molecular to macro levels, and focus on how advanced AI models can improve the accuracy of prediction.

### 3.1 Performance prediction of polymer biomaterials based on sequence and structure

The properties of biological materials, especially those based on natural macromolecules such as proteins, are usually closely related to their molecular sequences and multi-hierarchical structures. Traditional molecular dynamics simulation, while capable of predicting structural details, are computationally expensive and unsuitable for designing large-scale or complex systems (Wang et al., 2025e). Conversely, AI techniques have attained accurate predictions of the performance of biological polymers derived from proteins, peptides, and nucleic acids by examining the intricate mapping relationships among sequence, structure, and function.

The breakthrough lies in predicting protein structure and function through DL. For example, SeqPredNN, a feedforward neural network, had been proposed (Lategan et al., 2023). It had been trained on a database of X-ray crystal structures of proteins for prediction of types of amino acids in proteins solely based on the relative positions, orientations, and dihedral angles of nearby residues, effectively solving the “inverse protein folding problem”. In structural analysis, Wang et al. employed supervised ML to analyze circular dichroism spectra and related structural properties of 112 proteins, achieving more accurate predictions of complex secondary structures beyond typical α-helix and β-sheet (Wang and Kenry, 2025).

The primary sequence of proteins or nucleic acids significantly influences the properties of biomaterials. The COLOR model employs a DL model with interpretable steps to directly track the contribution of monomers to protein properties (Pandey et al., 2025). This model is 22% more interpretable than gradient and attention-based models in identifying key functional motifs. Deep neural network (DNN) is a potent AI model that can automatically learn and combine complex abstract features layer by layer from the original data, thereby achieving breakthrough performance in complex pattern recognition tasks such as image recognition and natural language processing. In the field of nucleic acids, AptaTrans, as a DNN pipeline, accurately predict the interaction between nucleic acid aptamers and proteins by leveraging a Transformer-based pre-trained encoder (Shin et al., 2023).

**FIGURE 2:**
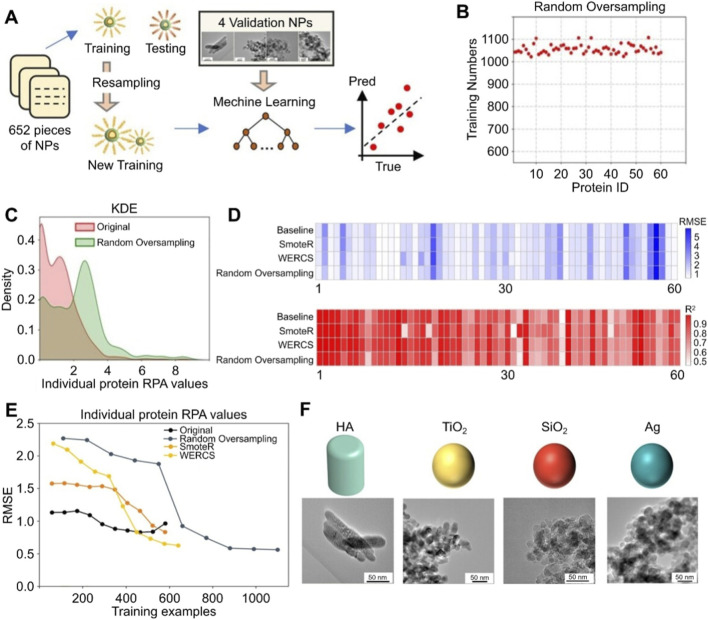
AI prediction model for the protein corona components of nanoparticles. **(A)** Flowchart for predicting protein corona using resampling embedding and RF model. **(B)** The number of training instances for 60 proteins after random oversampling. **(C)** The kernel density estimation plot of the first protein after random oversampling. **(D)** The root mean square error and R^2^ of 60 proteins. **(E)** The root mean square error curve of the RF model. **(F)** Four types of model nanoparticles. This Figure originated from Liao et al. (2023), Regenerative Biomaterials under CC BY 4.0 license. https://creativecommons.org/licenses/by/4.0/. Abbreviations: HA, hydroxyapatite; KDE, kernel density estimation; NPs, nanoparticles; RF, Random Forest; RMSE, root mean square error; RPA, relative protein abundance.

Moreover, predicting phenomena of complex biointerface also relies on a deep understanding of molecular behavior. After entering the bloodstream, nanoparticles will rapidly adsorb a shell composed of proteins and other biomolecules on their surface, known as a protein corona (Canchola et al., 2025). The protein corona endows nanoparticles with new properties, such as new identity tags or targeting capabilities. Accurate prediction of the protein crown composition will lay the foundation for revealing potential new properties of nanoparticles. For instance, Liao et al. addressed the issue of data imbalance by introducing resampling embedding technology and combining it with models such as random forest (RF), achieving accurate prediction of the protein corona composition on the surface of nanoparticles (Figure 2A) (Liao et al., 2023). After the implementation of data resampling technology, the relative protein abundance distribution of the protein corona was significantly improved (Figure 2B). In the kernel density estimation graph, the irregular low-density data points decreased and a single peak appeared (Figure 2C). After resampling, the RF model showed the root mean square error (RMSE) and R^2^ of 60 amino acids (Figure 2D). Compared with the baseline, the RMSE of the data processed by random oversampling decreased by 0.11, and the R^2^ increased by 0.06. As the sample size exceeded 600, the RMSE gradually stabilized at a lower level, proving the stability of the model (Figure 2E). Finally, the author selected four types of model nanoparticles and seven target proteins, and demonstrated that the model achieved predictive performance with an R^2^ value greater than 0.70 (Figure 2F).

### 3.2 Intelligent prediction of the physical properties of macroscopic biomaterials

For macroscopic biomaterials such as hydrogels and scaffolds, their physical properties including mechanical parameters, rheological characteristics, and degradation capabilities are the key factors determining their application effects (Tran et al., 2025). AI models significantly simplify material property characterization by establishing quantitative relationships between material composition, process parameters, and these physical properties.

Precise modeling of mechanical and rheological properties is one of the most widely applied fields. A study established an interpretable ML framework, XGBoost, based on a comprehensive dataset of 350 data points from existing literature to predict the tensile strain of polyvinyl alcohol (PVA) hydrogels, and achieved excellent prediction performance with test set R^2^ of 0.801 (Xu et al., 2025). The XGBoost model has also been utilized in predicting the mechanical properties of various material systems.

XGBoost illustrated the cumulative impact of the ratio between alginate dialdehyde and gelatin hydrogel, pore size, and the content of bioactive glass filler on the hardness of the hydrogel (Ege and Boccaccini, 2024). Assisted by ML algorithms such as XGBoost and AdaBoost, AI technology achieved highly accurate predictions of the mechanical properties of polylactic acid/calcium hydroxyapatite bone implants (Omigbodun et al., 2024). The R^2^ values for predicting compressive and tensile strength reached 0.9173 and 0.8772, respectively, highlighting the effectiveness of AI-driven methods in forecasting material properties. A study utilized 28 ML models to predict the Young’s modulus and ultimate tensile strength of PVA electrospun scaffolds, a category of tissue engineering implants, and effectively identified scaffold topologies that corresponded with the mechanical properties of real tissues (Roldán et al., 2024). This study indicated that the classification and regression trees model effectively identified the structure of biomimetic materials, whereas the cubist and support vector machine models were more adept at predicting the mechanical properties of materials.

In electrostatic spinning, the artificial neural network (ANN) model shows better efficiency in deriving algorithm functions from a limited experimental data set than the Box-Behnken design or non-neural network algorithm, so that it can more accurately predict the fiber diameter and tensile strength (Badaraev et al., 2025). ANN models were employed to precisely forecast the biodegradation rate, compressive strength, and hardness of tricalcium phosphate biological ceramics (Zilin et al., 2025). These parameters are crucial for the porosity of orthopedic implants and the ability to promote bone growth. DNN based on real experimental data was used to predict the stress-strain response of porous PVA/gelatin hydrogels under compressive loads (Khalvandi et al., 2023). Classification model, employing DNN was used to predict the ultimate tensile strength of silk fibers from amino acid sequences with an accuracy of 0.83. It was the first time that the macroscopic mechanical properties of biomaterials had been predicted with reference to the primary structure sequence (Shin et al., 2024).

In terms of rheological properties, researchers measured the rheological properties of different formulations of hyaluronic acid methacrylate/GelMA hydrogels, generated a dataset for training ML, and successfully established an AI model HydroThermoMLP for predicting the viscosity and shear stress of hydrogels (Deng et al., 2025).

The degradation performance and drug release curve of drug-loaded biomaterials are important parameters that determine the biocompatibility and efficacy. The Gaussian process regression model was used to predict the drug release curve of acetylated glucan nanofibers, demonstrating a method for predicting release kinetics without physical objects (Woodring et al., 2025). The gradient boosting regression model combined with leveraging firefly optimization had been proven to predict the release kinetics of controlled-release drugs in porous polymer carriers most effectively, with an R^2^ score of 0.9977 (Alshahrani et al., 2024).

Hydrogel-type biomaterials with high porosity usually have better biocompatibility and higher water retention capacity, so the prediction of porosity is of great significance. The gradient boosted regression tree model had been proven to be capable of accurately predicting poly (2-hydroxyethyl methacrylate)-poly (vinyl alcohol) composite (Wu et al., 2024). The effective porosity of hydrogel had a predicted percentage error of only 0.85%. The ANN model coupled with genetic algorithm was specially developed for accurately predicting the porosity of alginate gel scaffolds, and the mean absolute error reached 0.13 (Das et al., 2023).

In addition, some advanced AI models are used to predict other macroscopic properties of materials, such as fiber diameter and printability. Golbabaei et al. encoded various polymers through the simplified molecular input line entry system and combined with ML to achieve a prediction accuracy rate of 94.78% for the fiber diameter of electrospinning scaffolds (Golbabaei et al., 2024). The printability of 3D bioprinting ink denotes its capacity to be efficiently extruded, shaped, and retain its form during the 3D printing process, serving as a critical criterion for assessing the efficacy of 3D printing (Roppolo et al., 2024). Various ML techniques were employed to assess the printability of bioprinting inks according to their constituents (Chen et al., 2023). Among them, RF exhibited the highest performance, achieving a prediction accuracy of 88.1% and a precision of 90.6%. Rafieyan et al. developed a fully connected neural network by adjusting the hyperparameters of more than 40 AI algorithms, ranging from clustering analysis to DL, to accurately evaluate the printability of raw materials and the quality of scaffolds (Rafieyan et al., 2024). In addition, the Bayesian optimization framework was used to predict the viscosity of bioink precursors under constraints, and its external properties such as printability and biocompatibility were predicted through the mask function (Xu et al., 2024). Bayesian optimization, a design strategy used for optimizing black-box functions utilizes probabilistic models such as Gaussian processes as surrogate functions to predict the target, thereby finding the optimal solution with a very small sample size.

### 3.3 Application of advanced AI models in prediction of biomaterial properties

The forecasting of material properties by conventional AI algorithms is characterized as “black-box” prediction, with its primary issue stemming from the lack of explainability due to the intricate and obscure internal decision-making processes of the model. Nonetheless, the advent of rising AI technologies has elevated the accuracy and reliability of performance prediction by incorporating physical principles and elucidating the underlying mechanisms of the forecasts (Wong et al., 2025).

Physics-informed neural networks (PINNs) are a method that embeds physical equations into the neural network training process, enhancing the model’s generalization capability under data-scarce conditions. For instance, with the support of fundamental physical laws, PINNs simultaneously calculated the spatial distribution of an organization’s elastic modulus and Poisson’s ratio at one time (Kamali et al., 2023). Real experimental data and finite element modeling have verified the accuracy of the model, which is expected to be used to predict the mechanical properties of biomaterials implanted *in vivo* in real time. The DL model combined with the fractional order Lejande wavelet method can accurately predict the thermal behavior of biological tissue (Panigrahi et al., 2025). The experimental results show that the model reduces the thermal prediction error to 2.5 °C, and the calculation speed is 15% faster than the traditional method. It is very suitable for predicting the performance of biomaterials used in thermal therapy or photothermal therapy. ML-assisted finite element modeling technology using PINNs algorithm can accurately determine modeling parameters, so as to achieve accurate simulation of the macromechanical behavior of 3D printed biomaterials (Meynen et al., 2025). A model with two exponential linear fourth-order invariants is considered to be the best prediction model of the three-dimensional anisotropic relationship of warped polypropylene fabric (McCulloch and Kuhl, 2024). This model can be directly applied to other fabrics, or used to design programmable textile supermaterials.

The combination of DL and graph theory in complex structural analysis provides a new perspective for understanding the intricate relationship between microstructure and performance (Wang et al., 2016). For example, the open-source DL tool Pore D2 can automatically and accurately measure the diameter of each aperture or window in the scanned electron microscope image. This not only eliminates the expenses associated with manual measurement but also clearly presents the microscopic morphology of tissue engineering scaffolds (Karaca and Aldemir Dikici, 2024). ML-driven image analysis applied CNN to automatically recognize, segment and quantify nanoscale D-banding collagen fiber patterns in atomic force microscopy images, with an accuracy rate as high as 99% (Huang et al., 2025a). Zhong et al. integrated four types of staining and multi-dimensional parameters, constructed a semi-quantitative scoring standard and trained an AI model. The model increased the accuracy rate of residual cell nucleus recognition in acellular matrix biomaterials to over 98%, effectively eliminating false positives of impurities (Zhong et al., 2025). ML combined with visible and near-infrared spectroscopy had achieved non-destructive prediction of glycosaminoglycans and DNA content in tissue-engineered cartilage constructs, thereby assessing their maturity with an accuracy of 100% (Elkadi et al., 2025). The quantitative analysis of protein adsorption on 208 polymer microarrays was achieved by combining ML with liquid extraction surface analysis and tandem mass spectrometry, and a predictable ML model was generated, clarifying the relationship between the surface chemical properties of polymer-based biomaterials and their protein adsorption capacity (Meurs et al., 2025). There was also a rapid extraction method based on ML for automatically prediction of the three-dimensional orientation distribution of nanofiber biomaterials from wide-angle X-ray diffraction patterns (Sun et al., 2023).

An AI-driven predictive algorithm, integrating perturbation theory and neural networks, analyzed a dataset of over 1,200 bioprinting tests (Bediaga-Bañeres et al., 2025). The predictive algorithm achieved high-precision prediction of various properties of 3D-printed bioinks, demonstrating 88.4% specificity and 86.2% sensitivity.

In a word, AI has developed a comprehensive and intelligent biomaterial performance prediction framework. The framework first analyzes the molecular sequence and arrangement, then evaluates their physical and chemical properties, and finally uses advanced models based on physical laws to clarify complex structures. This innovative method has significantly shortened the research and development cycle of biomaterials, reduced costs, and laid a solid technical foundation for the design of the next-generation of high-performance biomaterials to meet specific needs.

## 4 Optimizing the material formula and processing technology

For biological materials for clinical applications, they not only need to show excellent performance to meet the challenges posed by complex medical scenarios, but also need to have repeatable formulas and production methods (Zhang et al., 2026). Traditional material optimization techniques, such as single-factor experiments, often find it difficult to deal with complex nonlinear interactions between various components and parameters involved in the design of biological materials. In contrast, AI models, especially through the integration of ML and optimization algorithms, can independently determine the best scheme for material synthesis, thus significantly improving the efficiency and effect of material formula and manufacturing process optimization.

### 4.1 Intelligent 3D printing technology and advanced manufacturing processes

Additive manufacturing technology, especially 3D printing and bioprinting, can manufacture biological materials with complex structures. However, the quality of printing and the performance of the final product will be significantly affected by many process parameters (Tian et al., 2025). AI models play a crucial role in optimizing the manufacturing process. They can establish an accurate mapping relationship between process parameters and performance results, so as to achieve the best adjustment and finally achieve the expected effect.

In optimizing the 3D printed bone support, a study adopted an integrated multimodal strategy, which combines the Taguchi L27 orthogonal table, the backpropagation ANN model and finite element analysis. This method systematically evaluates the effects of different lattice geometry, wall thickness and applied load on the displacement and strain of the bracket (Shetty et al., 2025). The research results not only highlight the excellent mechanical integrity of the spiral structure under specific stress conditions, but also develop a reverse propagation ANN model, which can predict the bracket displacement with an R^2^ value of up to 0.9991. This establishes a reliable calculation framework for enhancing the mechanical properties of 3D printed bone supports and improving the bone healing effect. Another study focused on polylactic acid bone support, and carefully examined the effects of nozzle temperature, printing speed and feed rate on Young’s modulus. Researchers introduced the ANN model to further optimize the printing parameters (Quan et al., 2025). Under the premise of satisfying the geometric constraints, the maximum Young’s modulus predicted by the ANN model is equivalent to the Young’s modulus of the loose bone, indicating that the stent optimization model has the potential to meet specific clinical needs.

In order to enhance the mechanical properties of polyvinyl alcohol (PVA) hydrogel scaffolds, researchers carefully examined four different preparation methods, all of which use PVA with a molecular weight of 145 kDa and a concentration gradient of 10–20 wt% (Li et al., 2025b). The quantitative analysis of the XGBoost ML model shows that the effect of the preparation process on the mechanical properties of hydrogel is as high as 74%, which is significantly higher than the impact of other parameters. It is worth noting that the compression modulus of the annealed hydrogel is 26 times higher than that of the hydrogel prepared by the traditional freeze-thaw method, and the friction coefficient is only 0.05. This example provides a clear process and scientific basis for the construction and optimization of high-strength pure PVA biomaterials. In another study, researchers used genetic algorithms to optimize the performance of palm fruit as a natural material (Samuel et al., 2024). The results show that the heating temperature of 50 °C combined with the insulation time of 120 min can make the tensile strength of the material twice that of the control group. This proves the potential of enhancing the mechanical properties of biomaterials through heat treatment, and provides valuable insights for engineering applications.

By fine-tuning the local characteristics of the printing structure, 3D printing biological materials can be further optimized. In order to accurately replicate the mechanical heterogeneity in real tissues (such as muscle tendon junctions (MTJ)) *in vitro*, researchers used AI training algorithms to optimize printing parameters (Kiratitanaporn et al., 2024b). They used 3D printing technology based on digital light processing to make different areas in poly (glyceryl decanoate) acrylate brackets. These areas are carefully designed with different stiffness, simulating the characteristics of muscles, tendons and tendon-muscle junctions respectively. The results of the experiment are encouraging. The scaffold shows the regional mechanical heterogeneity closest to the natural tendon junction, which significantly enhances the expression of molecular markers related to the tendon junction. This clearly shows the feasibility of using AI-assisted printing technology to build complex biological bionic design, opening up a new way for tissue engineering and regenerative medicine. Another study employed a neural network model to improve printing parameters, allowing the poly (glycerol sebacate) acrylate scaffold produced by light-curing 3D printing to be changed within a stiffness range of 49 kPa to 2.8 MPa (Kiratitanaporn et al., 2024a). This neural network model was compatible with both digital light processing and two-photon polymerization, enabling its application across a wide range of photopolymerization printing methods from the hundred-micrometer scale to the submicrometer scale. It provided scaffolds for tissues with mechanically gradient or heterogeneous interface structures.

Real-time monitoring of the component quality of 3D bioprinting materials to ensure the structural integrity is crucial for the optimization and continuous upgrading of the materials. A CNN model cropped confocal layer-by-layer images into small blocks, thereby identifying defect categories on hydrogels in real time and locating their spatial coordinates, with an accuracy rate of over 95% (Jin et al., 2023). This model can be directly embedded in the printer to achieve millisecond-level feedback closed loop, automatically correcting extrusion pressure, light intensity or path offset. The approach lays the foundation for optimizing the morphology and biocompatibility of tissue engineering constructs.

### 4.2 AI-driven optimization of material formulation and performance

In addition to the manufacturing process, the composition of the biomaterials is a fundamental factor that influences its performance. Through the analysis of the intricate interaction between components and performance, AI suggests the ideal material ratio, thus minimizing laborious trial-and-error tests.

**FIGURE 3:**
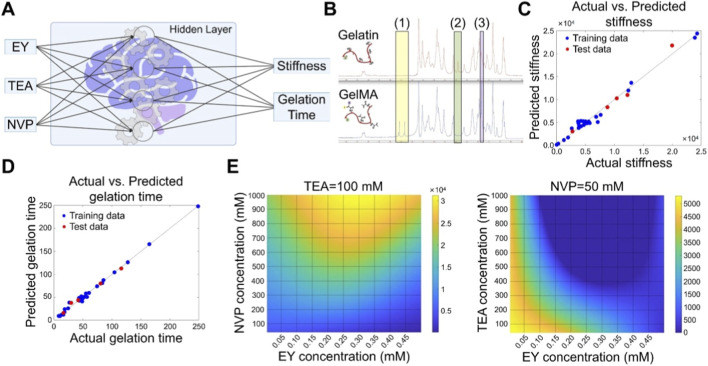
AI-driven optimization of optical cross-linking system for construction of GelMA hydrogel. **(A)** Scheme for ANN model which outputs the stiffness and gelation time through the proportion of EA, TEA and NVP. **(B)** ^1^H NMR spectra of gelatin and GelMA. **(C)** The linear correlation between the predicted and actual values of stiffness. **(D)** The linear correlation between the predicted and actual values of gelation time. **(E)** The library for Yong’s modulus of GelMA hydrogel established through the ANN model. Copyright 2023, American Chemical Society. Abbreviations: ANN, artificial neural network; EA, eosin Y; NVP, N-vinyl-2-pyrrolidone; TEA, triethanolamine.

Multiple studies have demonstrated the effectiveness of AI in optimizing hydrogel-based biomaterial systems. To construct an optimized paradigm for GelMA hydrogel, Karaoglu et al. systematically quantified the effects of concentrations of eosin Y (EY), triethanolamine (TEA), and n-vinyl-2-pyrrolidone (NVP) in the visible light cross-linked system on the modulus and gelation time (Figure 3A) (Karaoglu et al., 2023). Firstly, ^1^H NMR spectra proved the successful synthesis of GleMA (Figure 3B). EY, TEA and NVP were respectively set at five different levels, with a total of 125 formulas. Subsequently, the parameters of 125 hydrogels were detected to train an ANN model, which achieved an accuracy of 98.4% in the prediction of stiffness and 99.5% in the prediction of gelation time (Figures 3C,D). Based on this model, a comprehensive virtual database was established to predict the rigidity and gelation time of hydrogels with unknown formulations. For instance, after the concentration of TEA was fixed, the gel stiffness showed a significant increasing trend as the dosage of NVP increased, while in most cases, the gel stiffness was relatively low after the NVP was fixed (Figure 3E). This model provides a standardized paradigm for optimizing clinical-grade GelMA hydrogels. When developing new biomaterial scaffolds for tissue engineering, some studies combined the response surface methodology and ANN to optimize the natural polysaccharide scaffold composed of four components such as chitosan (Chinta et al., 2025). The optimal formula containing 36.1% chitosan was ultimately identified. The scaffold fabricated through this formula had a compressive strength of 0.4 MPa suitable for cartilage and showed good adhesion to various cells, providing an optimized platform for natural polysaccharid-based tissue engineering scaffolds.

The optimization method is also applicable to other material systems. A study had incorporated shear rate into the viscosity prediction of alginate-based ternary bioinks and established 169 sets of rheological data-driven ML models (Sarah et al., 2025). RF significantly outperformed decision tree and polynomial fitting with R^2^ of 0.99, achieving precise prediction from formulation to shear rate and viscosity and optimizing extruded bioprinting ink without experiments. 125 sets of polycaprolactone/polyethylene glycol electrospinning formulations and the corresponding material characterization data were used to train an ANN model (Virijević et al., 2024). The optimal electrospinning scaffold recommended by this model was adopted and used to deliver antibiotics around animal wounds. The density of new blood vessels increased by 2.3 times within 7 days, and the wound closure rate was 96% within 14 days, providing an expandable blueprint for the intelligent design of pro-healing scaffolds.

In the optimization of functional material, AI not only assist in optimizing the composition and formula of materials, but also directly enhance the performance of products at the application level. A patch with polyacrylic acid as the skeleton was used to prepare a stretchable strain flexible sensor (Liu et al., 2025). After optimizing the flexible sensing strategy through DL, the real-time recognition accuracy rate of the patch material for finger and wrist movements reached 99.33%, providing a preparation solution for self-adhesive, high-toughness wearable health monitoring device and flexible electronic skin. When developing natural lignocellulose dressing-based biological dressings, researchers utilized ML and human-computer interaction technologies to prepare new types of dressings that sensitively decoded multiple signals such as pressure and humidity in real time (Li et al., 2026).

### 4.3 Discovery of new biomaterials and high-throughput development

The ultimate potential of AI is in its capacity to transcend the constraints of human experience, autonomously investigate the extensive domains of chemistry and structure, uncover novel biological materials, or maximize the performance of existing materials.

Self-evolutionary discovery systems represent the cutting edge of this direction. To develop carriers for single-cell proteomics analysis, Hu et al. employed nine automated workstations in conjunction with an augmented Bayesian algorithm (Hu et al., 2025). This approach enabled self-iteration within the ultra-high-dimensional formulation space of carrier materials, yielding a super-inert surface with an 80% reduction in non-specific protein adsorption index compared to conventional methods after just over a hundred experimental cycles. The experimental workload was reduced by four orders of magnitude. The microfluidic chip obtained from the aforementioned carrier was used for single-cell protein analysis, and the detection sensitivity was increased by 9 times. This was the first verification that the AI-assisted self-evolution discovery system provided a universal carrier platform with high sensitivity for single-cell multi-omics.

In the design of new biopolymers, breakthroughs have been made in the integration of DL and bioengineering. To overcome the challenge of difficult heterologous expression of spider silk protein in prokaryotic systems, a study replaced the polyalanine of spider silk protein MASP1 with a bacterial sequence mined through DL, successfully achieving high expression of five soluble spider silk proteins in *Escherichia coli* (Huang et al., 2025b). These spider silk proteins can be electrospun into uniform nanofibers, providing a blueprint for the large-scale production of high-performance biomimetic fibers. PolypeptideDesigner, an AI model for polypeptide design, embedded an attention neural network as a denoiser within a conditional diffusion framework to generate novel polypeptides residue by residue (Liao et al., 2025). Compared to the industry-standard PDG-B model, PolypeptideDesigner designed longer and more diverse sequences, providing a powerful tool for the on-demand customization of functional peptides and biomaterials.

High-throughput screening and optimization is another key path to accelerate material discovery. To screen out uniform spheres from bioprinted models that can be used for liver transplantation, an automatic label-free sorter that can be stuffed into a biosafety cabinet was manufactured (Sampaio da Silva et al., 2024). This sorter employed transfer learning to rapidly distinguish activity and printability of multicellular spheres in brightfield images, achieving a sorting throughput of 600 spheroids per hour, thereby overcoming a critical bottleneck in tissue engineering therapies for end-stage liver disease. In the field of biofilm countermeasures, researchers had coupled individual biofilm models with Bayesian optimization to select optimal nanostructured surfaces tailored to specific applications (Zhai and Yeo, 2023). The selected nanoscale surface topography removed over 90% of biofilms, providing an on-demand surface design framework for fields requiring biofilm control, such as marine, medical, and bioenergy applications.

In a word, AI has become an indispensable catalyst for promoting the development of biological material formulas and processing methods. It can perform well in complex multi-parameter environments, enabling AI models to precisely control complex manufacturing processes such as 3D printing. This accuracy makes it possible to customize the transformation of biological materials from macro to micro levels. AI can not only recommend the most suitable material formula, but also independently discover new high-performance biological materials. AI has significantly shortened the time required for functional biomaterials to go from conceptual design to practical clinical application, thus opening a new era in the field of biomaterials.

## 5 Evaluating the effect of biomaterials on organisms

The effectiveness of biological materials depends not only on their physical and chemical properties, but more importantly on the complex biological reactions they cause when they interact with organisms. By integrating a variety of data types such as images, spectra and mechanical properties, these models can predict biological results before implantation, thus accelerating the development of safe and effective clinically applied biological materials.

### 5.1 Precise assessment and prediction of the biocompatibility of biomaterials

Once the biomaterial is implanted in the body, it will closely combine with the host cell, inevitably triggering the recognition and response of the immune system. Biocompatibility stems from the complex interaction between biomaterials and the host’s immune system after implantation. The quality of biocompatibility determines whether biomaterials are suitable for long-term use in the human body. Traditional biocompatibility assessment methods, such as cytotoxicity tests and hemolysis tests, can only provide basic information about these interactions. AI technology can interpret and predict the whole interaction process in real time, thus changing biocompatibility assessment from passive endpoint detection to active prediction.

**FIGURE 4:**
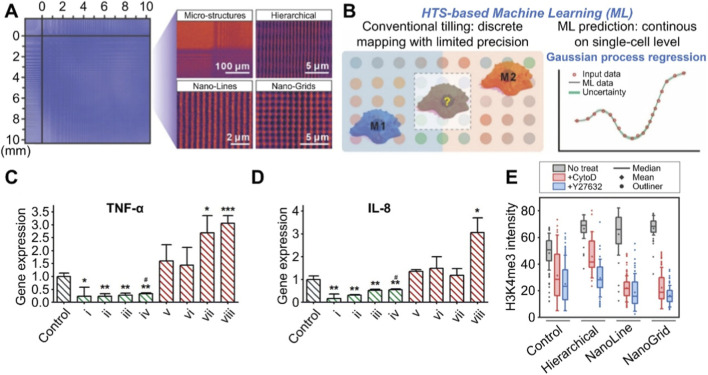
AI-driven prediction of macrophage polarization and inflammatory response to surface topological structures of implants. **(A)** Combined biophysical clue array of four structures. **(B)** Comparison of phenotypic prediction of macrophages using traditional method and ML. **(C)** The regulation of TNF-α expression level in macrophage by various nanostructures. **(D)** The regulation of IL-8 expression level in macrophage by various nanostructures. **(E)** The intensity of epigenetic activity marker H3K4me3 under conditions of no treatment, +cytotoxin and +Y27632. Copyright 2024, American Chemical Society. Abbreviations: IL-8, interleukin-8; ML, machine learning; TNF-α, tumor necrosis factor-α. All statistical data are represented as mean ± SD (**P < 0.05, **P < 0.01, ***P < 0.001).

Macrophages, as key regulators of the immune system, play a crucial role in tissue integration through their polarization states (Chen et al., 2025b). A study has established a label-free macrophage subtyping platform based on quantitative phase imaging and the DL model ResNet-18 (Martkamjan et al., 2025). This platform achieved an identification accuracy of over 90% for various macrophage phenotypes, including M0, M1, M2a, and M2c. It also revealed in real time the immune propensities of different collagen coatings—type I collagen is pro-inflammatory, while type IV collagen is anti-inflammatory, providing a novel AI-guided, rapid, and non-destructive paradigm for evaluating the immunocompatibility of biomaterials. Assessing the inflammatory response at the implant-tissue contact interface by examining the impact of surface topography on macrophage polarization is of crucial importance for the design and widespread application of implantable biomaterials. To establish a comprehensive nanoscale biointerface library through high-throughput screening, Hou et al. employed their self-developed dynamic laser interference lithography technique to fabricate, in a single batch, over one million types of line, grid, and hierarchical structures spanning scales from 100 nm to several micrometers, forming an array of combinatorial biophysical cues (Figure 4A) (Hou et al., 2024). After obtaining macrophage phenotypes, a Gaussian process regression ML model was employed to rapidly identify topological structures within the array that induced either M1 or M2 phenotypes (Figure 4B). *In vitro* experiments confirmed that nanostructures promoting the M1 macrophage phenotype indeed enhanced the expression of pro-inflammatory genes, including tumor necrosis factor-α (TNF-α) and interleukin-8 (IL-8), whereas M2-biased nanostructures exhibited the opposite regulatory effects (Figures 4C,D). Further mechanistic studies revealed that, compared to the control group, three nanostructures respectively increased macrophage transcriptional levels by 1.38-fold, 1.38-fold, and 1.29-fold, indicating that these three topological structures might regulate macrophage polarization via epigenetic activation, particularly involving the cytoskeleton and Rho-associated protein kinases (Figure 4E). These results demonstrated that this AI-based screening model could swiftly pinpoint immunomodulatory active topological structures from a vast array of morphologies at the million-scale, providing a reliable standard for the subsequent mass production of nano-coatings for implants.

There are also studies exploring the influence of various physical parameters of the implant on the polarization of macrophages. Based on over 1,200 experimental data points from more than 30 research articles, Chen et al. developed algorithmic rules that establish correlations between parameters such as contact angle and roughness of titanium-based implants and the levels of interleukin-10 (IL-10) and TNF-α secreted by macrophages, utilizing models including RF, extreme gradient boosting, and multi-layer perceptron (Chen et al., 2025a). The algorithm demonstrated an error margin of less than 8% in predicting IL-10 levels, proving that this model offered an interpretable and reproducible AI design paradigm for immunomodulatory implants. Similarly, a study employed various ML approaches to train and analyze the inflammatory responses of macrophages induced by 15 types of electrospun nanofibrous scaffolds composed of polyesters, polysaccharides, and polyethers (Sujeeun et al., 2025). In these models, the prediction accuracy of TNF-α levels by random forests (RF) reached 92.8%, and it was found that fiber-oriented entropy and polysaccharide percentage were the key parameters affecting macrophage inflammation.

When evaluating the biological safety of materials, it is very important to accurately predict the toxicity of biological materials to tissue cells. A study developed 5 ML models using 51 pure zinc cytotoxicity data sets (Wang et al., 2025a). The multilayer perceptron (MLP) model shows that the survival rate of bone cells, endothelial cells and fibroblasts is high when the leachate concentration does not exceed 40%; the decision tree model also proves that the leachate concentration is a key predictor variable. The study determined that 40% was the *in vitro* toxicity threshold of zinc-based implants, and created a standardized, machine-based machine-learning-based toxicity assessment system for biodegradable zinc-based implants. Another study evaluated the prediction accuracy of the DL model for the biocompatibility of tissue scaffolds. The results showed that when using structured numerical data, the ANN model was better than the CNN model, and its F1 score reached 1.0 (Oncu et al., 2025).

At present, the definitions and evaluation standards for biocompatibility are different in different fields, which hinders data standardization and its integration with AI models. Mozafari proposed a method to redefine biocompatibility through a unified “hierarchical modular” architecture (Mozafari, 2025). This method systematically evaluates biological materials based on clinical background, material classification and exposure time, so that AI models can access structured big data. This structured method accelerates material discovery, regulatory approval and post-listing risk monitoring, and promotes the data-driven progress of biomaterial development.

### 5.2 Dynamic assessment of tissue regeneration and functional formation

The main biological function of biological materials is to promote the regeneration and functional recovery of normal tissues. AI technology has significantly improved our understanding of the process of organization generation through non-destructive monitoring, early prediction and dynamic evaluation.

In the field of cartilage regeneration monitoring and evaluation, a study used near-infrared spectrum combined with non-biased ML algorithms (such as random forests and support vector regression) for the first time to monitor the supernatant of the engineered cartilage culture system. The expected R^2^ values of hyaluronic acid, lactic acid and total collagen are 0.98, 0.70 and 0.44 respectively, which provides a sampling-free method for real-time quality assessment of synthetic cartilage (Sadeesh et al., 2025). Another study shows that the near-infrared spectrum combined with the ResNet-18 model has achieved an identification accuracy of more than 90% in distinguishing between normal cartilage regions and degenerative cartilage regions, which helps to early evaluate cartilage degradation in a microgravity environment (Wu et al., 2025). In the field of bone tissue engineering, a research has developed a descending model based on ML, which is combined with Shapley’s additional interpretation to predict the degree of osteogenic differentiation (Drakoulas et al., 2024). Meanwhile, it was discovered that the scaffold stiffness of 200–250 MPa was most conducive to osteogenesis. After optimizing the multi-objective genetic algorithm based on this conclusion, the bone cell coverage area increased from 38% to 61%, providing a novel paradigm for prediction and design of 3D-printed biodegradable scaffolds. In the aspect of vascularization assessment, scholars have designed an AI-based software named IKOSA (Salvante et al., 2024). It utilized the chorioallantoic membrane CAM model and microscopic images to evaluate vascular area, lumen length, and vascular branching potential. Based on this research, an AI pre-screening platform had been proposed, which may replace mammalian experiments for the rapid assessment of the vascularization potential of biomaterials.

AI technologies exhibit significant benefits in the analysis of intricate tissue imaging data and the development of high-throughput screening platforms. A study employed cleared tissue light-sheet microscopy to acquire three-dimensional autofluorescence maps of whole organs and volumetric muscle defect specimens at an isotropic resolution of 0.6 μm (Ngo et al., 2023). By utilizing computational spectral classification, this method, for the first time, achieved three-dimensional analysis of the interaction microenvironment between host tissue and implanted scaffolds at the single-cell scale.

### 5.3 Early prediction and manipulation of cell behavior and fate

AI technology enables the prediction of long-term cell fates based on early cell morphology and behavior, serving as a potent instrument for the swift evaluation of biomaterial biological performance.

In the field of immune cell therapy, it was found that the subsequent mechanosensitive expansion potential of T cells can be predicted based on their short-term spreading morphology on biomaterials. A DL model distinguished between healthy and chronic lymphocytic leukemia donors with an accuracy of over 92% using only 6-h bright-field images (Wang et al., 2025d). Moreover, it predicted the cell expansion fold on substrates of different stiffnesses 4 weeks in advance with an error of less than 15%, providing a rapid and precise quality prediction tool for adoptive cellular immunotherapy. Another automated ML-based detection method first employed thresholding and cell tracking to swiftly delineate candidate cell division events (Manorost et al., 2025). Subsequently, it utilized ANOVA testing to screen for eight-dimensional interpretable features. Finally, it uses RF algorithms to eliminate artifacts. This method achieved an impressive accuracy of 88%, striking a balance between speed and interpretability. Compared to DL, it was more suitable for the automated monitoring of high-density cell proliferation.

For the prediction of stem cell fate, Zhou et al. integrated public transcriptome databases and combined the k-nearest neighbor strategy to develop a three-line differentiation prediction framework for mesenchymal stem cells (Zhou et al., 2023). This framework determined the differentiation direction of human mesenchymal stem cells on various biomaterials on the seventh day without the need for markers. It achieved a testing accuracy of 90.6%, which is 10% higher than that of traditional marker gene method, providing a rapid assessment tool for high-throughput screening of material biological functions. Another AI model, the orthopedic implants-osteogenic differentiation network, utilized intuitive early-stage cell morphology images and alkaline phosphatase levels as training data. It enabled high-throughput prediction of the osteogenic potential of titanium surfaces within 48 h, enhancing the screening speed of orthopedic implant surface layers by 10-fold and reducing costs by 80% (Li et al., 2025a). To achieve label-free cell differentiation assessment and material screening, Hao et al. constructed a light-controlled thiol-ene high-throughput chip (Hao et al., 2023). The device can generate 128 different material combinations in one operation. They use unmarked CNN combined with classification and statistical models to evaluate the surface of materials with specific biological functions.

A study assessing gene therapy efficacy involved the implantation of fibroblasts derived from patients with Leber hereditary optic neuropathy and subjected to AAV gene transduction, into a three-dimensional artificial scaffold (Larin et al., 2024). The study demonstrated that a DL algorithm model designed to monitor and analyze cellular characteristics such as migration speed, proliferation rate, and morphology, could potentially replace animal testing in evaluating drugs targeting genetic cellular disorders.

AI has fundamentally changed the way we evaluate the impact of biological materials on organisms, and established a comprehensive and multi-dimensional evaluation framework covering from cell phenotype to tissue function. This paradigm shift not only accelerates the clinical transformation of safe and effective biological materials, but also fundamentally changes the way we understand and develop these materials.

## 6 Discussion

This article discusses how AI affects the design process of biomaterials, and puts forward an AI-based intelligent closed-loop paradigm. The paradigm advocated in this review represents a dynamic, iterative, and self-improving system where AI seamlessly integrates each stage of biomaterial development. The loop initiates with AI-driven reverse design based on clinical needs, whose outputs are fed into predictive models to forecast properties and screen candidates virtually. The optimal candidates then undergo AI-guided optimization of their formulation and manufacturing process. Finally, the loop closes with the AI-powered evaluation of biological responses, whose results—such as newly discovered structure-activity relationships or unsatisfied biological performance—can be fed back to refine the initial design goals or prediction models. This creates a virtuous cycle where data and insights from downstream stages continuously enhance the upstream design, ultimately accelerating the development toward high-performance, clinically viable biomaterials.

As we enter this new era, we must realize that there are many major problems in the current AI system. Future development depends on how effectively we deal with these challenges.

The main problem lies in the quality and quantity of data. Biomaterial data, including composition, processing and performance, come from various sources, resulting in differences between batches. The lack of a standardized database limits the generalization ability of AI models (Mozafari, 2025). High-quality experimental data, especially long-term *in vivo* biological data, are expensive and scarce, making model training difficult. In order to solve this problem, the field of biomaterials should establish standardized data collection and reporting specifications, and create an open and standardized data sharing platform. Considering that the data may involve confidential information, a federal ML model can be used to allow cross-agency joint training models while protecting data privacy and expanding the training sample size (Montagnese et al., 2025).

The second problem is the inherent limitations of the AI model. Despite tools such as SHapley Additive exPlanations (SHAP) analysis, the decision-making logic of many complex models, especially DL models, is still unclear (Xu et al., 2025). It is difficult for researchers to explain why the model makes certain predictions, which hinders the model from providing reliable physical or biological insights for the discovery of new materials. In addition, when applied to new material systems or cell types, AI models usually perform poorly and are less robust. To solve this problem, the existing AI model should integrate explainable AI technologies, such as attention mechanism and counter-factual interpretation, to identify key factors and reveal mechanisms, so as to accelerate scientific discovery (Zhou et al., 2023). It is also crucial to develop a neural network based on the principles of physics that embeds physical laws into model algorithms, which can ensure that the prediction results are in line with scientific principles (Naga Ramesh et al., 2025). Furthermore, the model reproducibility is also a major obstacle to the reliable adoption of AI technology. This means that even if the same algorithm is used, differences in experimental procedures and data processing can still lead to vastly different results. Therefore, the use of containerized computing environments, such as Docker are recommended to ensure the consistency of model performance across different laboratories and datasets (Hernández-Velázquez et al., 2025).

Thirdly, biomaterials operate across multiple scales from molecules and cells to tissues and organs. However, most current AI models are designed for a single scale and lack a framework that integrates physical, chemical, and biological processes across scales. To overcome this, multi-scale AI models have been proposed, which combine molecular dynamics simulations, mesoscopic models, and macroscopic models using AI to predict the entire lifecycle of biomaterials from preparation to *in vivo* use.

AI is driving a significant shift in the biomaterial field, moving from a supplementary tool to the core of the research and development process. A future of fully integrated, automated, and personalized intelligent biomaterial development is on the horizon. In the future, researchers may only need to input clinical requirements into a computer, and the AI system will autonomously generate material solutions, predict their performance, operate robotic platforms for synthesis and optimization, and evaluate their biological effects. To achieve this, enhanced interdisciplinary collaboration among biologists, materials scientists, doctors, and AI specialists is needed. We believe AI will become a powerful ally in fighting diseases, promoting tissue healing, and improving human health.

## References

[B1] Alshahrani S. M. Alotaibi H. F. Begum M. Y. (2024). Computational analysis of controlled drug release from porous polymeric carrier with the aid of mass transfer and artificial intelligence modeling. Sci. Rep. 14, 28422. 10.1038/s41598-024-79749-6 39558051 PMC11574292

[B2] Badaraev A. D. Rutkowski S. Davoodi S. Tverdokhlebov S. I. (2025). Predicting diameter and tensile strength of electrospun fibers for biomedicine: a comparison of box-behnken design, traditional machine learning and deep learning. Comput. Biol. Med. 196, 110923. 10.1016/j.compbiomed.2025.110923 40803173

[B3] Bediaga-BañERES H. Moreno-BeníTEZ I. Arrasate S. PéREZ-Álvarez L. Halder A. K. Cordeiro M. (2025). Artificial intelligence-driven modeling for hydrogel three-dimensional printing: computational and experimental cases of study. Polym. (Basel) 17, 121. 10.3390/polym17010121 39795524 PMC11723248

[B4] Cadamuro F. Piazzoni M. Gamba E. Sonzogni B. Previdi F. Nicotra F. (2025). Artificial intelligence tool for prediction of ECM mimics hydrogel formulations via click chemistry. Biomater. Adv. 175, 10. 10.1016/j.bioadv.2025.214323 40315575

[B5] Canchola A. Li K. Chen K. Borboa-Pimentel A. Chou C. Dela Rama R. (2025). Meta-analysis and machine learning prediction of protein Corona composition across nanoparticle systems in biological media. ACS Nano 19, 37633–37650. 10.1021/acsnano.5c08608 41031442 PMC12593346

[B6] Chen H. Liu Y. Balabani S. Hirayama R. Huang J. (2023). Machine learning in predicting printable biomaterial formulations for direct ink writing. Research (Wash D C), 6, 0197. 10.34133/research.0197 37469394 PMC10353544

[B7] Chen C. Xie Z. Yang S. Wu H. Bi Z. Zhang Q. (2025a). Machine learning approach to investigating macrophage polarization on various titanium surface characteristics. BME Front. 6, 0100. 10.34133/bmef.0100 40012846 PMC11862448

[B8] Chen R. Q. Liu P. J. Li S. He H. P. Li D. M. Yuan G. X. (2025b). Healing of tendon-related diseases: insights from macrophage regulation. Mil. Med. Res. 12, 45. 10.1186/s40779-025-00635-x 40760449 PMC12320333

[B9] Chinta M. L. Gandam P. K. Parcha S. R. (2025). Design and optimization of tamarind seed polysaccharide-based scaffold for tissue engineering applications using statistical modeling and machine learning, and it's *in-vitro* validation. Int. J. Biol. Macromol. 301, 140411. 10.1016/j.ijbiomac.2025.140411 39889978

[B10] Cho Y. Beak J. W. Sagong M. Ahn S. Nam J. S. Kim I. D. (2025). Electrospinning and nanofiber technology: fundamentals, innovations, and applications. Adv. Mater 37, e2500162. 10.1002/adma.202500162 40190229 PMC12272013

[B11] Das R. Karthika S. Bhasarkar J. Bal D. K. (2023). GA-coupled ANN model for predicting porosity in alginate gel scaffolds. J. Mech. Behav. Biomed. Mater 148, 106204. 10.1016/j.jmbbm.2023.106204 37883894

[B12] Deng B. Chen S. Lasaosa F. L. Xue X. Xuan C. Mao H. (2025). Predicting rheological properties of HAMA/GelMA hybrid hydrogels via machine learning. J. Mech. Behav. Biomed. Mater 168, 107005. 10.1016/j.jmbbm.2025.107005 40228459

[B13] Drakoulas G. Gortsas T. Polyzos E. Tsinopoulos S. Pyl L. Polyzos D. (2024). An explainable machine learning-based probabilistic framework for the design of scaffolds in bone tissue engineering. Biomech. Model Mechanobiol. 23, 987–1012. 10.1007/s10237-024-01817-7 38416219

[B14] Ege D. Boccaccini A. R. (2024). Investigating the effect of processing and material parameters of alginate dialdehyde-gelatin (ADA-GEL)-Based hydrogels on stiffness by XGB machine learning model. Bioeng. (Basel) 11, 415. 10.3390/bioengineering11050415 38790283 PMC11117982

[B15] ElkadiO. A. AbinzanoF. NippolainenE. GonzáLEZO. B. TöYRäSJ. LevatoR. (2025). Non-destructive assessment of tissue engineered cartilage maturity using visible and near infrared spectroscopy combined with machine learning. Biosens. Bioelectron. 286, 117587. 10.1016/j.bios.2025.117587 40413993

[B16] Fang Z. Zhang M. Wang H. Chen J. Yuan H. Wang M. (2023). Marriage of high-throughput gradient surface generation with statistical learning for the rational design of functionalized biomaterials. Adv. Mater 35, e2303253. 10.1002/adma.202303253 37795620

[B17] Ghafarollahi A. Buehler M. J. (2025). Automating alloy design and discovery with physics-aware multimodal multiagent AI. Proc. Natl. Acad. Sci. U. S. A. 122, e2414074122. 10.1073/pnas.2414074122 39854228 PMC11789045

[B18] Golbabaei M. H. Varnoosfaderani M. S. Hemmati F. Barati M. R. Pishbin F. Seyyed Ebrahimi S. A. (2024). Machine learning-guided morphological property prediction of 2D electrospun scaffolds: the effect of polymer chemical composition and processing parameters. RSC Adv. 14, 15178–15199. 10.1039/d4ra01257g 38737974 PMC11082644

[B19] Hang R. Yao X. Bai L. Hang R. (2025). Evolving biomaterials design from trial and error to intelligent innovation. Acta Biomater. 197, 29–47. 10.1016/j.actbio.2025.03.013 40081552

[B20] Hao H. Xue Y. Wu Y. Wang C. Chen Y. Wang X. (2023). A paradigm for high-throughput screening of cell-selective surfaces coupling orthogonal gradients and machine learning-based cell recognition. Bioact. Mater 28, 1–11. 10.1016/j.bioactmat.2023.04.022 37214260 PMC10192934

[B21] HernáNDEZ-VeláZQUEZ R. Ziemski M. Bokulich N. A. (2025). ViromeXplore: integrative workflows for complete and reproducible virome characterization. Brief. Bioinform 26, bbaf638. 10.1093/bib/bbaf638 41348596 PMC12862488

[B22] Hou Y. Conklin B. Choi H. K. Yang L. Lee K. B. (2024). Probing nanotopography-mediated macrophage polarization via integrated machine learning and combinatorial biophysical cue mapping. ACS Nano 18, 25465–25477. 10.1021/acsnano.4c04406 39226301 PMC13003755

[B23] Hu S. Lu W. Ding X. Xue Y. Liu C. Xie T. (2025). Self-evolving discovery of carrier biomaterials with ultra-low nonspecific protein adsorption for single cell analysis. Adv. Mater 37, e2506243. 10.1002/adma.202506243 40605415

[B24] Huang S. Ng N. Vaez M. Hinz B. Leong I. Bozec L. (2025a). Collagen hybridizing peptides promote collagen fibril growth *in vitro* . ACS Appl. Bio Mater 8, 2003–2014. 10.1021/acsabm.4c01509 40010706 PMC11921027

[B25] Huang Y. Qi J. An B. Zhang B. Yang Y. Cheng C. (2025b). High-yield spidroin mimics for bioinspired fibers via computational design. Front. Bioeng. Biotechnol. 13, 1587546. 10.3389/fbioe.2025.1587546 40343205 PMC12058734

[B26] Jia H. Zhang H. Mo D. Xie B. Qiao H. Chen T. (2025). UTX responds to nanotopography to suppress macrophage inflammatory response by remodeling H3K27me3 modification. Adv. Sci. (Weinh) 12, e05723. 10.1002/advs.202505723 40386877 PMC12362724

[B27] Jiang Z. H. Feng J. W. Wang F. Wang J. K. Wang N. T. Zhang M. M. (2025). AI-Guided design of antimicrobial peptide hydrogels for precise treatment of drug-resistant bacterial infections. Adv. Mater. 37, 20. 10.1002/adma.202500043 40159831

[B28] Jin Z. Zhang Z. Shao X. Gu G. X. (2023). Monitoring anomalies in 3D bioprinting with deep neural networks. ACS Biomater. Sci. Eng. 9, 3945–3952. 10.1021/acsbiomaterials.0c01761 33882674

[B29] Kamali A. Sarabian M. Laksari K. (2023). Elasticity imaging using physics-informed neural networks: spatial discovery of elastic modulus and Poisson's ratio. Acta Biomater. 155, 400–409. 10.1016/j.actbio.2022.11.024 36402297 PMC9805508

[B30] Karaca I. Aldemir Dikici B. (2024). Quantitative evaluation of the pore and window sizes of tissue engineering scaffolds on scanning electron microscope images using deep learning. ACS Omega 9, 24695–24706. 10.1021/acsomega.4c01234 38882138 PMC11170757

[B31] Karaoglu I. C. Kebabci A. O. Kizilel S. (2023). Optimization of gelatin methacryloyl hydrogel properties through an artificial neural network model. ACS Appl. Mater Interfaces 15, 44796–44808. 10.1021/acsami.3c12207 37704030

[B32] Khalvandi A. Tayebi L. Kamarian S. Saber-Samandari S. Song J. I. (2023). Data-driven supervised machine learning to predict the compressive response of porous PVA/gelatin hydrogels and *in-vitro* assessments: employing design of experiments. Int. J. Biol. Macromol. 253, 126906. 10.1016/j.ijbiomac.2023.126906 37716655

[B33] Kiratitanaporn W. Guan J. Berry D. B. Lao A. Chen S. (2024a). Multimodal three-dimensional printing for micro-modulation of scaffold stiffness through machine learning. Tissue Eng. Part A 30, 280–292. 10.1089/ten.TEA.2023.0193 37747804

[B34] Kiratitanaporn W. Guan J. Tang M. Xiang Y. Lu T. Y. Balayan A. (2024b). 3D printing of a biomimetic myotendinous junction assisted by artificial intelligence. Biomater. Sci. 12, 6047–6062. 10.1039/d4bm00892h 39446075

[B35] Larin I. I. Shatalova R. O. Laktyushkin V. S. Rybtsov S. A. Lapshin E. V. Shevyrev D. V. (2024). Deep learning for cell migration in nonwoven materials and evaluating gene transfer effects following AAV6-ND4 transduction. Polym. (Basel) 16, 1187. 10.3390/polym16091187 38732656 PMC11085928

[B36] Lategan F. A. Schreiber C. Patterton H. G. (2023). SeqPredNN: a neural network that generates protein sequences that fold into specified tertiary structures. BMC Bioinforma. 24, 373. 10.1186/s12859-023-05498-4 37789284 PMC10546711

[B37] Lehman J. (2025). AI discovers learning algorithm that outperforms those designed by humans. Nature 648, 283–285. 10.1038/d41586-025-03398-6 41125918

[B38] Li A. Li X. Zhang Z. Huang Z. He L. Yang Y. (2025a). Deep learning assisted prediction of osteogenic capability of orthopedic implant surfaces based on early cell morphology. Acta Biomater. 195, 559–568. 10.1016/j.actbio.2025.01.059 39894326

[B39] Li J. Zhang C. Lan W. Chen W. Huang D. (2025b). Machine learning-assisted strategies to enhance the mechanical properties of PVA hydrogels. J. Mech. Behav. Biomed. Mater 168, 107027. 10.1016/j.jmbbm.2025.107027 40273621

[B40] Li C. Du J. Zhu L. Hu J. Fu C. Lu J. (2026). Natural lignocellulose fibers-based bio-dressing for accelerated wound healing and machine learning-assisted smart multimodal sensing. Biomaterials 325, 123603. 10.1016/j.biomaterials.2025.123603 40784131

[B41] Liao R. Zhuang Y. Li X. Chen K. Wang X. Feng C. (2023). Unveiling protein Corona composition: predicting with resampling embedding and machine learning. Regen. Biomater. 11, rbad082. 10.1093/rb/rbad082 38213739 PMC10781662

[B42] Liao S. Xu G. Jin L. Ma J. (2025). *De novo* Design of Large Polypeptides Using a Lightweight Diffusion Model Integrating LSTM and Attention Mechanism Under Per-Residue Secondary Structure Constraints. Molecules 30, 1116. 10.3390/molecules30051116 40076339 PMC11902264

[B43] Liu S. Wang Z. Wei Q. Duan X. Liu Y. Wu M. (2023). Biomaterials-enhanced bioactive agents to efficiently block spinal metastases of cancers. J. Control Release 363, 721–732. 10.1016/j.jconrel.2023.09.039 37741462

[B44] Liu S. Shi J. Liu D. Wang H. Xiong J. Du Z. (2025). A flexible and adhesive strain sensor based on deep eutectic solvents for deep learning-assisted signal recognition. ACS Appl. Mater Interfaces 17, 27076–27091. 10.1021/acsami.4c20392 40274546

[B45] Manorost P. Deckers T. Bloemen V. Aerts J. M. (2025). Explainable handcrafted features for mitotic event detection and classification. Sci. Rep. 15, 7382. 10.1038/s41598-025-87180-8 40025100 PMC11873241

[B46] Martkamjan C. Lerdsudkanung K. Tipay P. S. Rezgui R. Teo J. C. M. Sapudom J. (2025). Machine learning-based label-free macrophage phenotyping in immune-material interactions. J. Mater Chem. B 13, 5858–5870. 10.1039/d5tb00365b 40289902

[B47] Mcculloch J. A. Kuhl E. (2024). Automated model discovery for textile structures: the unique mechanical signature of warp knitted fabrics. Acta Biomater. 189, 461–477. 10.1016/j.actbio.2024.09.051 39368719

[B48] Meurs J. Nasir A. Figueredo G. P. Burroughs L. Abdelrazig S. A. Denning C. (2025). High-throughput analysis of protein adsorption to a large library of polymers using liquid extraction surface analysis-tandem mass spectrometry (LESA-MS/MS). Anal. Chem. 97, 12776–12785. 10.1021/acs.analchem.5c01636 40492276 PMC12199226

[B49] Meynen A. Kolken H. Mulier M. Zadpoor A. A. Scheys L. (2025). Machine learning-assisted finite element modeling of additively manufactured meta-materials. 3D Print Med. 11, 36. 10.1186/s41205-025-00286-7 40658303 PMC12257752

[B50] Montagnese M. Rangelov B. Doel T. Llewellyn D. Walker Z. Rittman T. (2025). Cloud computing for equitable, data-driven dementia medicine. Lancet Digit. Health, 100902. 10.1016/j.landig.2025.100902 41207814

[B51] Mozafari M. (2025). Artificial intelligence in biomaterials: a call for unified biocompatibility standards. Trends Biotechnol. 43, 266–267. 10.1016/j.tibtech.2024.11.011 39933892

[B52] Naga Ramesh J. V. Sonker A. Indumathi G. Balakrishnan D. Nimma D. Karthik J. (2025). Bayesian neural networks for probabilistic modeling of thermal dynamics in multiscale tissue engineering scaffolds. J. Therm. Biol. 130, 104134. 10.1016/j.jtherbio.2025.104134 40381543

[B53] Ngo T. B. Destefano S. Liu J. Su Y. Shroff H. Vishwasrao H. D. (2023). Label-free cleared tissue microscopy and machine learning for 3D histopathology of biomaterial implants. J. Biomed. Mater Res. A 111, 840–850. 10.1002/jbm.a.37515 36861434 PMC12316057

[B54] Omigbodun F. T. Osa-Uwagboe N. Udu A. G. Oladapo B. I. (2024). Leveraging machine learning for optimized mechanical properties and 3D printing of PLA/cHAP for bone implant. Biomimetics (Basel) 9, 587. 10.3390/biomimetics9100587 39451792 PMC11504968

[B55] Oncu E. Usta Ayanoglu K. Y. Ciftci F. (2025). Comparative analysis of deep learning models for predicting biocompatibility in tissue scaffold images. Comput. Biol. Med. 192, 110281. 10.1016/j.compbiomed.2025.110281 40306018

[B56] Pandey A. Chen W. Keten S. (2025). COLOR: a compositional linear operation-based representation of protein sequences for identification of monomer contributions to properties. J. Chem. Inf. Model 65, 4320–4333. 10.1021/acs.jcim.5c00205 40272990

[B57] Panigrahi B. S. Nath S. S. Agarwal P. Muthu Kumar B. Karimunnisa S. Neeladri M. (2025). Hybrid AI models for predicting heat distribution in complex tissue structures with bioheat transfer simulation. J. Therm. Biol. 129, 104122. 10.1016/j.jtherbio.2025.104122 40311397

[B58] Quan R. Cantero Chinchilla S. Liu F. (2025). Investigation of the effects of 3D printing parameters on the mechanical properties of bone scaffolds: experimental study integrated with artificial neural networks. Bioeng. (Basel) 12, 315. 10.3390/bioengineering12030315 40150779 PMC11939716

[B59] Rafieyan S. Ansari E. Vasheghani-Farahani E. (2024). A practical machine learning approach for predicting the quality of 3D (bio)printed scaffolds. Biofabrication 16, 045014. 10.1088/1758-5090/ad6374 39008994

[B60] RoldáN E. Reeves N. D. Cooper G. Andrews K. (2024). Machine learning to mechanically assess 2D and 3D biomimetic electrospun scaffolds for tissue engineering applications: between the predictability and the interpretability. J. Mech. Behav. Biomed. Mater 157, 106630. 10.1016/j.jmbbm.2024.106630 38896922

[B61] Roppolo I. Caprioli M. Pirri C. F. Magdassi S. (2024). 3D printing of self-healing materials. Adv. Mater 36, e2305537. 10.1002/adma.202305537 37877817

[B62] Sadeesh N. Karunarathna H. Jalali A. Oikari S. Eskelinen A. Gebraad A. (2025). Non-destructive monitoring of cartilage tissue engineering via near-infrared (NIR) spectroscopic assessment of culture medium. Biosens. Bioelectron. 288, 117809. 10.1016/j.bios.2025.117809 40695111

[B63] Salvante E. R. G. Popoiu A. V. Barb A. C. Cosma A. A. Fenesan M. P. Saxena A. K. (2024). Artificial intelligence (AI) based analysis of *in vivo* polymers and collagen scaffolds inducing vascularization. In Vivo 38, 620–629. 10.21873/invivo.13481 38418141 PMC10905450

[B64] Sampaio Da Silva C. Boos J. A. Goldowsky J. Blache M. Schmid N. Heinemann T. (2024). High-throughput platform for label-free sorting of 3D spheroids using deep learning. Front. Bioeng. Biotechnol. 12, 1432737. 10.3389/fbioe.2024.1432737 39717531 PMC11663632

[B65] Samuel B. O. Alabi A. A. Lawal S. A. Peter E. Ibrahim T. K. (2024). Optimizing the effect of heat treatment on the mechanical properties (tensile strength and hardness) of Hyphaene thebaica nut; A machine learning and taguchi approach. Heliyon 10, e38899. 10.1016/j.heliyon.2024.e38899 39430497 PMC11489375

[B66] Sarah R. Schimmelpfennig K. Rohauer R. Lewis C. L. Limon S. M. Habib A. (2025). Characterization and machine learning-driven property prediction of a novel hybrid hydrogel bioink considering extrusion-based 3D bioprinting. Gels 11, 45. 10.3390/gels11010045 39852017 PMC11765179

[B67] Shetty A. Fathima A. Anika B. Shetty R. Supriya J. P. Hegde A. (2025). Computational optimization of 3D printed bone scaffolds using orthogonal array-driven FEA and neural network modeling. Sci. Rep. 15, 30515. 10.1038/s41598-025-15122-5 40835669 PMC12368192

[B68] Shin I. Kang K. Kim J. Sel S. Choi J. Lee J. W. (2023). AptaTrans: a deep neural network for predicting aptamer-protein interaction using pretrained encoders. BMC Bioinforma. 24, 447. 10.1186/s12859-023-05577-6 38012571 PMC10680337

[B69] Shin H. Yoon T. You J. Na S. (2024). A study of forecasting the nephila clavipes silk fiber's ultimate tensile strength using machine learning strategies. J. Mech. Behav. Biomed. Mater 157, 106643. 10.1016/j.jmbbm.2024.106643 38945120

[B70] Song J. Dong J. Yuan Z. Huang M. Yu X. Zhao Y. (2024). Shape-persistent conductive nerve guidance conduits for peripheral nerve regeneration. Adv. Healthc. Mater 13, e2401160. 10.1002/adhm.202401160 38757919

[B71] Sujeeun L. Y. Phul I. C. Goonoo N. Kotov N. A. Bhaw-Luximon A. (2025). Predicting inflammatory response of biomimetic nanofibre scaffolds for tissue regeneration using machine learning and graph theory. J. Mater Chem. B 13, 3304–3318. 10.1039/d4tb02494j 39869000

[B72] Sun M. Dong Z. Wu L. Yao H. Niu W. Xu D. (2023). Fast extraction of three-dimensional nanofiber orientation from WAXD patterns using machine learning. IUCrJ 10, 297–308. 10.1107/s205225252300204x 36961758 PMC10161767

[B73] Sun Y. Yang L. Du L. Zhou Y. Xu K. Chen J. (2024). Duo-role Platelet-rich plasma: temperature-induced fibrin gel and growth factors' reservoir for microneedles to promote hair regrowth. J. Adv. Res. 55, 89–102. 10.1016/j.jare.2023.02.014 36849045 PMC10770113

[B74] Tian Z. Tsui G. C. Tang Y. M. Wong C. H. Tang C. Y. Ko C. C. (2025). Additive manufacturing for nanogenerators: fundamental mechanisms, recent advancements, and future prospects. Nanomicro Lett. 18, 30. 10.1007/s40820-025-01874-2 40788321 PMC12339862

[B75] Tran C. M. Yue Z. Qin C. Imani K. B. C. Dottori M. Forster R. J. (2025). 3D printing of conducting polymer hydrogels for electrostimulation-assisted tissue engineering. Adv. Mater 37, e2507779. 10.1002/adma.202507779 40714743 PMC12422089

[B76] Virijević K. Živanović M. N. Nikolić D. Milivojević N. Pavić J. Morić I. (2024). AI-Driven optimization of PCL/PEG electrospun scaffolds for enhanced *in vivo* wound healing. ACS Appl. Mater Interfaces 16, 22989–23002. 10.1021/acsami.4c03266 38659385

[B77] Wang Z. Kenry (2025). Machine-learning-guided identification of protein secondary structures using spectral and structural descriptors. Biomater. Sci. 13, 2973–2982. 10.1039/d5bm00153f 40297866

[B78] Wang R. Sing M. K. Avery R. K. Souza B. S. Kim M. Olsen B. D. (2016). Classical challenges in the physical chemistry of polymer networks and the design of new materials. Acc. Chem. Res. 49, 2786–2795. 10.1021/acs.accounts.6b00454 27993006

[B79] Wang H. Fu Y. Mao J. Jiang H. Du S. Liu P. (2022). Strong and tough supramolecular microneedle patches with ultrafast dissolution and rapid-onset capabilities. Adv. Mater 34, e2207832. 10.1002/adma.202207832 36189863

[B80] Wang Z. Dabaja R. Chen L. Banu M. (2023). Machine learning unifies flexibility and efficiency of spinodal structure generation for stochastic biomaterial design. Sci. Rep. 13, 5414. 10.1038/s41598-023-31677-7 37012266 PMC10070414

[B81] Wang Q. Gao C. Zhai H. Peng C. Yu X. Zheng X. (2024a). Electrospun scaffolds are not necessarily always made of nanofibers as demonstrated by polymeric heart valves for tissue engineering. Adv. Healthc. Mater 13, e2303395. 10.1002/adhm.202303395 38554036

[B82] Wang Y. Huang R. Lu Y. Liu M. Mo R. (2024b). Immuno-protective vesicle-crosslinked hydrogel for allogenic transplantation. Nat. Commun. 15, 5176. 10.1038/s41467-024-49135-x 38890279 PMC11189436

[B83] Wang Q. Chen C. Liu Q. Li Q. Chen J. Zhang Q. (2025a). Machine learning-based optimization of cytotoxicity testing for assessing Zn-based biodegradable metals. Mater Today Bio 32, 101816. 10.1016/j.mtbio.2025.101816 40487167 PMC12145565

[B84] Wang R. Li J. Bi Q. Yang B. He T. Lin K. (2025b). Crystallographic plane-induced selective mineralization of nanohydroxyapatite on fibrous-grained titanium promotes osteointegration and biocorrosion resistance. Biomaterials 313, 122800. 10.1016/j.biomaterials.2024.122800 39241551

[B85] Wang W. Li Z. Zhang S. Ma Y. Yu L. Zhang Q. (2025c). A pseudo-mytilus edulis foot protein-based hydrogel adhesive with osteo-vascular-immune coupling effects for osteoporotic bone-implant integration. Adv. Mater, e11840. 10.1002/adma.202511840 41190935 PMC12848646

[B86] Wang X. Xu R. Hu S. Sun D. Guo J. Lamanna N. (2025d). Predicting mechanosensitive T cell expansion from cell spreading. Adv. Healthc. Mater 14, e01925. 10.1002/adhm.202501925 40717390 PMC12367345

[B87] Wang Y. Li Y. Chen J. Lai L. (2025e). Modeling protein-ligand interactions for drug discovery in the era of deep learning. Chem. Soc. Rev. 54, 11141–11183. 10.1039/d5cs00415b 41117015

[B88] Wang Y. Song H. Teng Y. Huang G. Qian J. Wang H. (2025f). A generative artificial intelligence copilot for biomedical nanoengineering. ACS Nano 19, 19394–19407. 10.1021/acsnano.5c03454 40367350

[B89] Wong F. Omori S. Li A. Krishnan A. Lach R. S. Rufo J. (2025). An explainable deep learning platform for molecular discovery. Nat. Protoc. 20, 1020–1056. 10.1038/s41596-024-01084-x 39653800 PMC12668791

[B90] Woodring R. N. Gurysh E. G. Pulipaka T. Shilling K. E. Stiepel R. T. Pena E. S. (2025). Supervised machine learning for predicting drug release from acetalated dextran nanofibers. Biomater. Sci. 13, 2806–2823. 10.1039/d5bm00259a 40237176 PMC12553120

[B91] Wu J. Wang R. Tan Y. Liu L. Chen Z. Zhang S. (2024). Hybrid machine learning model based predictions for properties of poly(2-hydroxyethyl methacrylate)-poly(vinyl alcohol) composite cryogels embedded with bacterial cellulose. J. Chromatogr. A 1727, 464996. 10.1016/j.chroma.2024.464996 38763087

[B92] Wu Q. Ji X. Wu J. Li Y. Hui Z. Wang H. (2025). *In-situ* NIR spectroscopy study on microgravity-induced articular cartilage degeneration with ResNet-18. Spectrochim. Acta A Mol. Biomol. Spectrosc. 348, 127188. 10.1016/j.saa.2025.127188 41242109

[B93] Xu Y. Sarah R. Habib A. Liu Y. Khoda B. (2024). Constraint based Bayesian optimization of bioink precursor: a machine learning framework. Biofabrication 16, 045031. 10.1088/1758-5090/ad716e 39163881

[B94] Xu L. Liu S. Lin A. Su Z. Liang D. (2025). Interpretable prediction and analysis of PVA hydrogel mechanical behavior using machine learning. Gels 11, 550. 10.3390/gels11070550 40710711 PMC12294283

[B95] Yan P. Sun J. Zhao Y. Deng W. Zhang M. Li Y. (2025). Machine learning-driven optimization of therapeutic substance composition for high-hardness, fast-dissolving microneedles for androgenetic alopecia treatment. ACS Nano 19, 29301–29315. 10.1021/acsnano.5c05505 40782084

[B96] Yang L. Pijuan-Galito S. Rho H. S. Vasilevich A. S. Eren A. D. Ge L. (2021). High-throughput methods in the discovery and study of biomaterials and materiobiology. Chem. Rev. 121, 4561–4677. 10.1021/acs.chemrev.0c00752 33705116 PMC8154331

[B97] Zhai H. Yeo J. (2023). Computational design of antimicrobial active surfaces via automated bayesian optimization. ACS Biomater. Sci. Eng. 9, 269–279. 10.1021/acsbiomaterials.2c01079 36537745

[B98] Zhang J. Li J. Zhao G. Wang Q. Guo Y. G. Yang C. (2025a). Mining solid-state electrolytes from metal-organic framework databases through large language models and representation clustering. J. Am. Chem. Soc. 147, 40496–40506. 10.1021/jacs.5c12212 41131733

[B99] Zhang Y. Fu Y. Sun T. Li W. Yu L. Ding J. (2025b). Skin relevant biomaterials from wound healing, medical aesthetics, flexible electronics to artificial intelligence and beyond. Adv. Mater, e12919. 10.1002/adma.202512919 41025745 PMC13549263

[B100] Zhang C. Shen Y. Huang M. Wang G. Miao Q. Shi H. (2026). Dynamic hydrogel mechanics in organoid engineering: from matrix design to translational paradigms. Bioact. Mater 55, 144–170. 10.1016/j.bioactmat.2025.09.021 41035429 PMC12481939

[B101] Zheng B. Xie Y. Xu S. Meng A. C. Wang S. Wu Y. (2024). Programmed multimaterial assembly by synergized 3D printing and freeform laser induction. Nat. Commun. 15, 4541. 10.1038/s41467-024-48919-5 38806541 PMC11133382

[B102] Zhong M. He H. Ni P. Huang C. Zhang T. Chen W. (2025). Semi-quantitative scoring criteria based on multiple staining methods combined with machine learning to evaluate residual nuclei in decellularized matrix. Regen. Biomater. 12, rbae147. 10.1093/rb/rbae147 39886363 PMC11780845

[B103] Zhou Y. Ping X. Guo Y. Heng B. C. Wang Y. Meng Y. (2023). Assessing biomaterial-induced stem cell lineage fate by machine learning-based artificial intelligence. Adv. Mater 35, e2210637. 10.1002/adma.202210637 36756993

[B104] Zhou W. Liu Y. Dong J. Hu X. Su Z. Zhang X. (2024). Mussel-derived and bioclickable peptide mimic for enhanced interfacial osseointegration via synergistic immunomodulation and vascularized bone regeneration. Adv. Sci. (Weinh) 11, e2401833. 10.1002/advs.202401833 38922775 PMC11348244

[B105] Zhu Y. Yu X. Liu H. Li J. Gholipourmalekabadi M. Lin K. (2024). Strategies of functionalized GelMA-based bioinks for bone regeneration: recent advances and future perspectives. Bioact. Mater 38, 346–373. 10.1016/j.bioactmat.2024.04.032 38764449 PMC11101688

[B106] Zilin Z. Yun X. Baghei S. (2025). A new approach to bioceramics based on tissue reaction of tricalcium phosphate for biomedical and sport applications using machine learning modeling. Tissue Cell 95, 102899. 10.1016/j.tice.2025.102899 40188687

